# Supplementary material for: A low-cost benthic incubation chamber for in-situ community metabolism measurements
Source: PeerJ. 2022 Apr 5;10:e13116. doi: 10.7717/peerj.13116 (PMC8992662; doi:10.7717/peerj.13116)
Supplement: Supplemental Information 1 [file peerj-10-13116-s001.docx]

**Table S1:** Wilcoxon rank sum test with continuity correction to test differences between photosynthetically active radiation (PAR in µmol photons m^-2^ s^-1^) inside vs. outside chambers during deployments over 3 days in August 2019.

|  | Date measured | PAR  (mean ±SD in µmol m^-2^ s^-1^) | | W statistic | p-value |
| --- | --- | --- | --- | --- | --- |
|  |  | inside | outside |  |  |
| Full day (0700 to 1900) | 01/08/19 | 258 ±238 | 289 ±267 | 2322 | 0.3305 |
|  | 03/08/19 | 269 ±239 | 300 ±268 | 2151 | 0.418 |
|  | 07/08/19 | 216 ±255 | 242 ±285 | 2882 | 0.2474 |
| Solar peak only (1200 to 1400) | 01/08/19 | 461 ±195 | 515 ±218 | 86 | 0.4428 |
|  | 03/08/19 | 622 ±116 | 696 ±130 | 109 | 0.03324 |
|  | 07/08/19 | 611 ±22 | 684 ±25 | 144 | 7.396 x  10^-07^ |
